# *Lignosus rhinocerotis* extract ameliorates airway inflammation and remodelling via attenuation of TGF-β1 and Activin A in a prolonged induced allergic asthma model

**DOI:** 10.1038/s41598-023-45640-z

**Published:** 2023-10-27

**Authors:** Siti-Aminah Muhamad, Sabreena Safuan, Johnson Stanslas, Wan Amir Nizam Wan Ahmad, Solehah-Mohd-Rosdan Bushra, Asma Abdullah Nurul

**Affiliations:** 1 School of Health Sciences, Universiti Sains Malaysia, 16150 Kubang Kerian, Kelantan Malaysia; 2 Pharmacotherapeutics Unit, Department of Medicine, Faculty of Medicine and Health Sciences, Universiti Putra Malaysia, Serdang, Selangor Malaysia

**Keywords:** Drug discovery, Immunology, Diseases, Medical research

## Abstract

Allergic asthma is associated with chronic airway inflammation and progressive airway remodelling. The sclerotium of *Lignosus rhinocerotis* (Cooke) Ryvarden (Tiger Milk mushroom) is used traditionally to treat various illnesses, including asthma in Southeast Asia. This study was carried out to evaluate the effect of *L. rhinocerotis* extract (LRE) on airway inflammation and remodelling in a chronic model of asthma. The present study investigated the therapeutic effects of LRE on airway inflammation and remodelling in prolonged allergen challenged model in allergic asthma. Female Balb/C mice were sensitised using ovalbumin (OVA) on day 0 and 7, followed by OVA-challenged (3 times/week) for 2, 6 and 10 weeks. LRE (125, 250, 500 mg/kg) were administered by oral gavage one hour after every challenge. One group of mice were left untreated after the final challenge for two weeks. LRE suppressed inflammatory cells and Th2 cytokines (IL-4, IL-5 and IL-13) in BALF and reduced IgE level in the serum. LRE also attenuated eosinophils infiltration and goblet cell hyperplasia in the lung tissues; as well as ameliorated airway remodelling by reducing smooth muscle thickness and reducing the expressions of TGF-β1 and Activin A positive cell in the lung tissues. LRE attenuated airway inflammation and remodelling in the prolonged allergen challenge of allergic asthma model. These findings suggest the therapeutic potential of LRE as an alternative for the management of allergic asthma.

## Introduction

Asthma is a chronic respiratory disorder which characterised by pathophysiological symptoms such as hyperresponsiveness, obstruction, remodelling, and inflammation of the airway^1^. It is a serious public health problem affecting people of all ages^2^. More than 300 million people are affected globally, with at least 250,000 deaths each year, and its prevalence is increasing, especially in developing countries^3^. Hence presenting a major public health and socioeconomic burden^4^.

In asthma pathophysiology, a complex combination of genetic, environmental, and immunological factors play role. The pathogenesis and development of asthma are orchestrated by a structured relationship between lung structural cells including airway epithelium and components of the innate and adaptive immune systems^5, 6^. Allergic asthma manifested by recurrent episodes of airway inflammation composed of infiltrating eosinophils, mast cells, macrophages, neutrophils, and lymphocytes^1, 4, 5^ and the activated cells will release the pro-inflammatory mediators such as histamine and reactive oxygen species (ROS). They will induce the contraction of airways smooth muscle, promote mucous secretion and vasodilatation. During the inflammation process, IgE and mast cells are implicated in the acute response and T-helper 2 cells, orchestrating these responses through the production of cytokines. Multiple cytokines, chemokines, and growth factors are released from both inflammatory and structural cells in the airway tissue to create a complex signalling environment that promotes the development of airway remodelling.

Cytokines and activation of the inflammatory cascade contribute to both the allergic reaction and the remodelling of lung tissue. Airway remodelling in asthmatic patients is induced by increased infiltration of T lymphocytes and eosinophils^7^. Airway remodelling encompasses complex changes in composition, content, distribution, thickness and organisation of the various cellular and molecular constituents of the airway wall of asthmatic patients^8, 9^. The most striking abnormalities in the chronic airway inflammation are epithelial denudation, goblet cell metaplasia, sub-epithelial thickening, increased airway smooth muscle mass, bronchial gland enlargement, angiogenesis, and alterations in the extracellular matrix (ECM) components which are associated with irreversible loss of lung function^10^.

The current available medication for asthma treatment offers only symptomatic relief and has several limitations. Hence, new approaches to the management of asthma are required. Several reports have suggested that asthma can be managed by targeting the remodelling and inflammation of the airway^11^. Thus, a more effective alternative is needed, and natural products seem to be a promising approach. Meanwhile, there are increasing shreds of evidence that natural products demonstrated promising findings to be an alternative for the management of asthma^12–14^. Recently, unique properties of *L. rhinocerotis* have been explored and proven scientifically for various therapeutic effects. Previous studies have shown that *L. rhinocerotis* sclerotia exhibited immunomodulatory^15^, anti-proliferative^16^ and anti-inflammatory properties which were demonstrated by *L. rhinocerotis* in the carrageenan induced paw oedema model in rats^17–19^ reported the anti-asthmatic properties of *L. rhinocerotis* in an acute asthma model. These studies reported that *L. rhinocerotis* extract (LRE) exhibited anti-asthmatic properties by reducing the level of Th2 cytokines and IgE and alleviating the number of leukocyte infiltration in the lung tissues. To our knowledge, there is no study evaluating the anti-asthmatic effects of LRE in a chronic model of asthma. Herein, the aim of this study was to assess the effects of LRE on the airway inflammation and remodelling in a chronic model of asthma.

## Results

### Effects of LRE on inflammatory cells in prolonged OVA-challenged allergic asthma model

**Figure 1:**
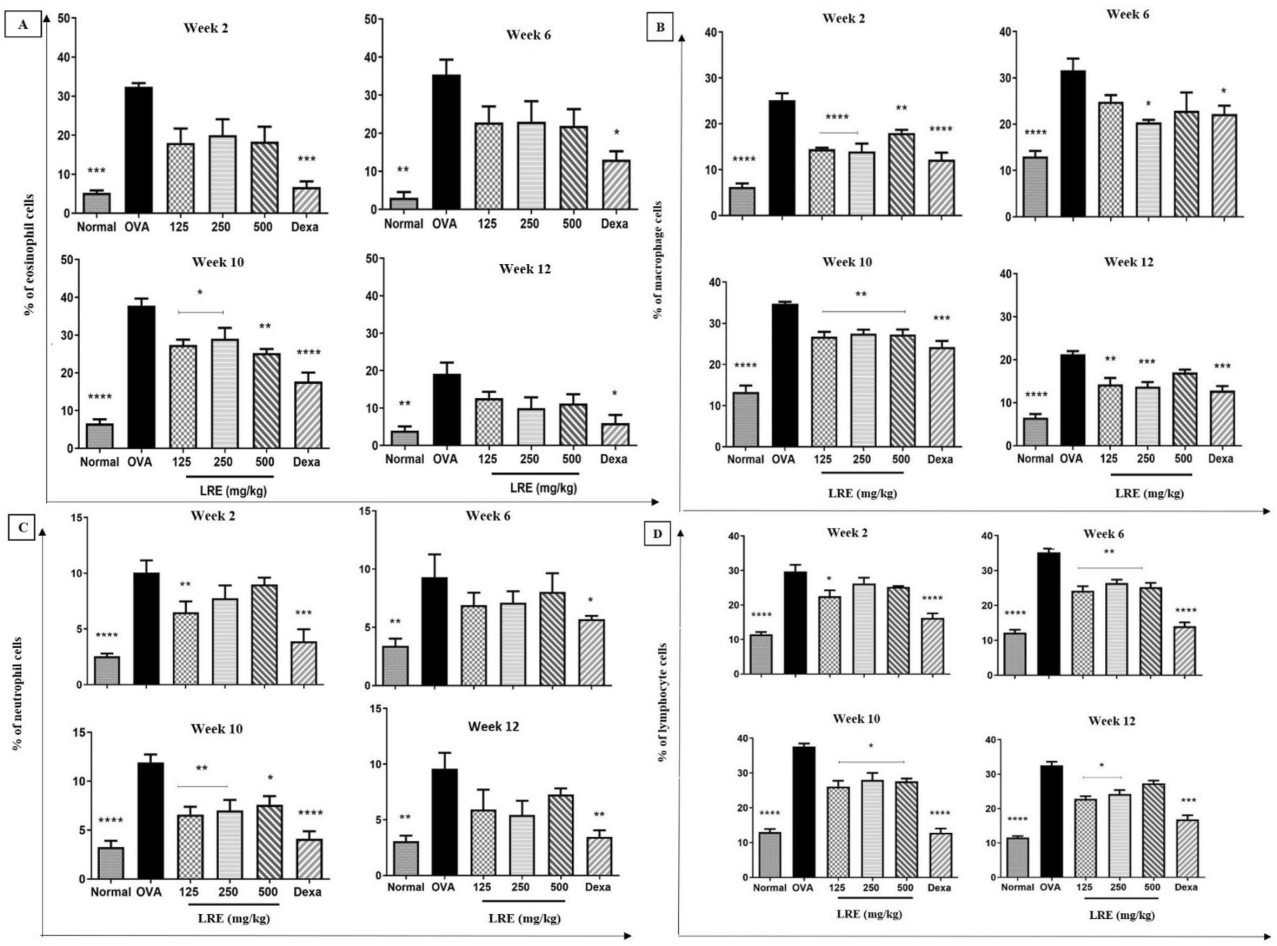
Effects of LRE on inflammatory cells recruitment in bronchoalveolar lavage in prolonged OVA-challenged mice. Normal; OVA sensitised/ challenged mice; LRE125 mg/kg + OVA-sensitised/challenged mice; LRE250 mg/kg + OVA- sensitised/challenged mice; LRE500 mg/kg + OVA-sensitised/ challenged mice; dexamethasone + OVA-sensitised/challenged mice. Values are expressed as means ± SEM (n = 7 per group). OVA-group is significantly different from the normal group. *p < 0.05, **p < 0.01, ***p < 0.001 and ****p < 0.0001 indicates significant difference from OVA.

During the prolonged allergen challenge, OVA induction significantly increased the percentage of inflammatory cells (eosinophil, macrophage, neutrophil and lymphocyte) in the BALF in comparison to the normal group. Figure 1 showed that treatment with dexamethasone significantly reduced the percentage of inflammatory cells following the prolonged OVA challenge. Meanwhile, LRE-treated groups (125, 250 and 500 mg/kg) showed a different pattern in attenuating the percentage of inflammatory cells. LRE-treated groups showed significant attenuation of eosinophils count during the Week 10. A similar attenuation was observed on the percentage of neutrophil cells by the LRE-treated groups on Week 10. Interestingly, consistent attenuations were observed by LRE250-treated group on the percentage of macrophage cells throughout the treatment durations. Meanwhile, LRE125-treated group showed a consistent attenuation for the lymphocyte, throughout the treatment durations.

### Effects of LRE on IL-4, IL-5, IL-13 and IgE level in prolonged OVA-challenged allergic asthma model

**Figure 2:**
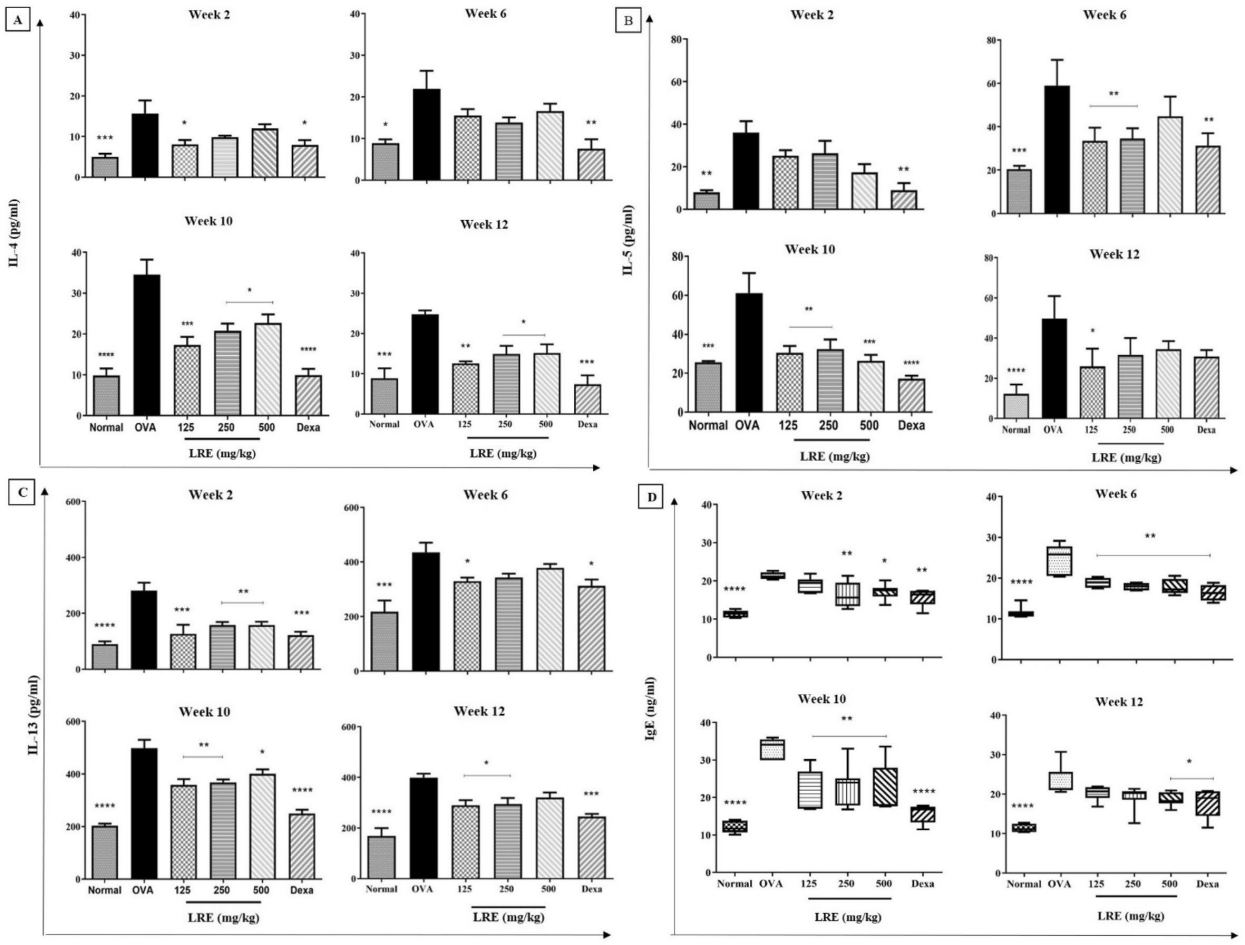
Effects of LRE on Th2 cytokines (IL-4, IL-5 and IL-13) production and IgE level in prolonged OVA-challenged mice. Normal; OVA sensitised/ challenged mice; LRE125 mg/kg + OVA-sensitised/challenged mice; LRE250 mg/kg + OVA- sensitised/challenged mice; LRE500 mg/kg + OVA- sensitised/challenged mice; dexamethasone + OVA-sensitised/challenged mice. Values are expressed as means ± SEM (n = 7 per group). OVA-group is significantly different from the normal group. *p < 0.05, **p < 0.01, ***p < 0.001 and ****p < 0.0001 indicates significant difference from OVA.

We highlighted the validity of our model through the significant increase of Th2 cytokines (IL-4, 5 and 13) in BALF and the level of IgE in serum. It is interesting to note that the levels of IL-4, IL-5, IL-13 and IgE showed a remarkable increase in OVA-challenged mice during prolonged allergen challenge (week 2, 6, 10 and 12) in comparison to normal group (Fig. 2). The level of IL-4 was significantly reduced in LRE-treated groups in week 10 and 12. During Week 2, a significant reduction of IL-4 was observed in LRE125-treated group; meanwhile, Dexa group showed a significant reduction of IL-4 level throughout the treatment durations. The level of IL-5 was significantly reduced in LRE125 and LRE250 in Weeks 6 and 10. A significant reduction of IL-5 was observed in the LRE125 group in Week 12, and Dexa group demonstrated a significant reduction of IL-5 level in Weeks 2, 6 and 10. The LRE-treated groups significantly reduced the level of IL-13 in Weeks 2 and 10. However, at week 6, only LRE125 showed a significant reduction of the IL-13 level. Meanwhile, after allergen cessation on week 12, LRE125 and LRE250 significantly reduced the level of IL-13. Dexa group showed a significant reduction of IL-13 level throughout the treatment durations. The level of IgE was significantly reduced by LRE250 and LRE500 after 2 weeks of the challenge. All the LRE-treated groups showed a significant reduction in IgE level during Weeks 6 and 10. In the absence of allergen challenge on Week 12, LRE500 significantly reduced the level of IgE.

### The effects of LRE on leukocyte infiltration in the lungs of prolonged OVA-challenged allergic asthma model

**Figure 3:**
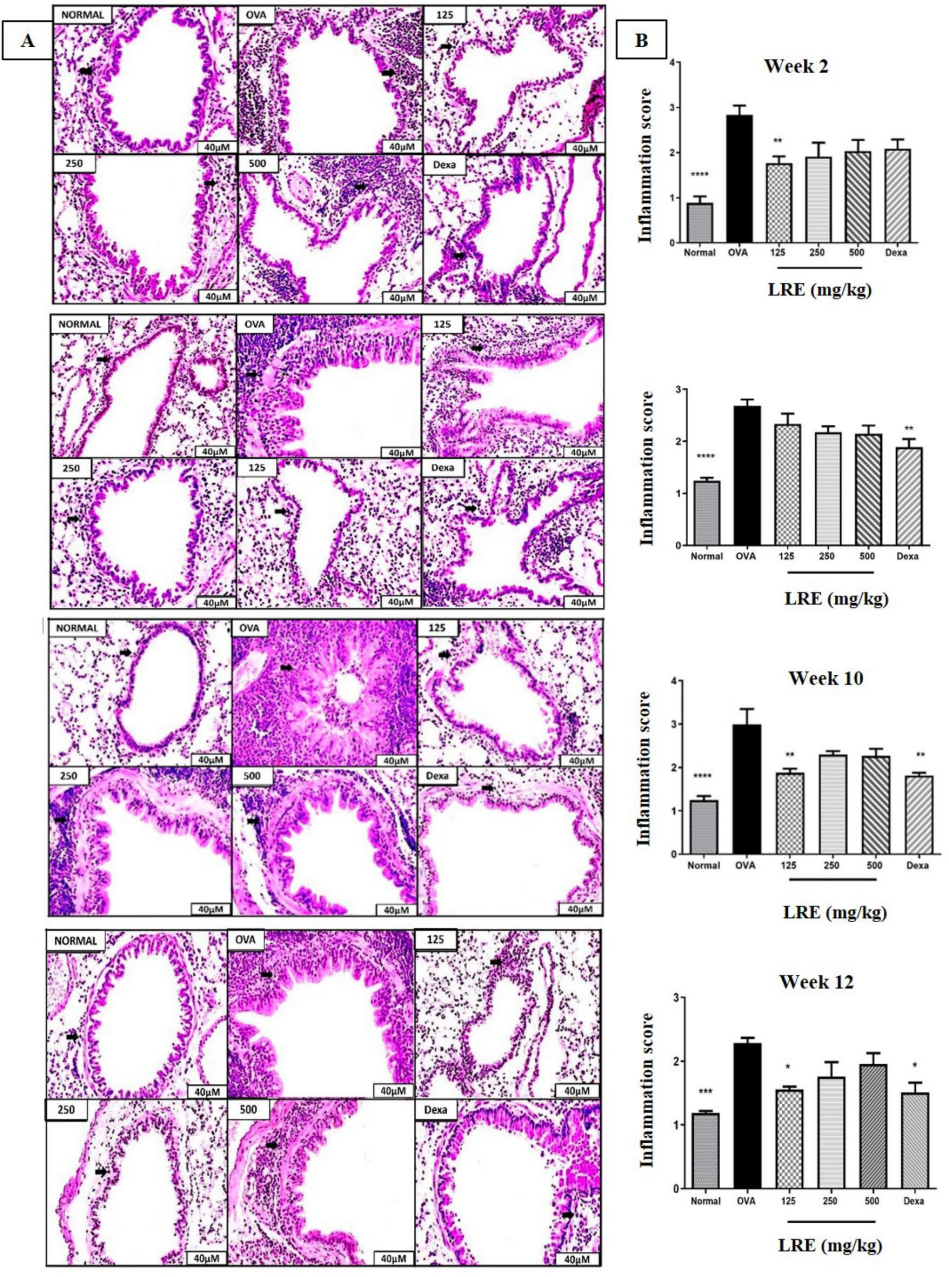
The effects of LRE on leukocytes infiltration in the lung tissue of prolonged OVA- challenged mice. (**A**) Representative photomicrographs OVA-sensitised/challenged mice; Normal; LRE125 mg/kg + OVA-sensitised/challenged mice; LRE250 mg/kg + OVA-sensitised/challenged mice; LRE500 mg/kg + OVA-sensitised/challenged mice; dexamethasone + OVA-sensitised/challenged mice. Black arrows indicate the presence of inflammatory cell infiltrate surrounding the bronchiole. All figures are in 40 × magnification. (**B**) Semi-quantitative analysis on inflammation score with a subjective scale of 0–4. Values are expressed as means ± SEM (n = 7 per group). OVA-group is significantly different from the normal group. *p < 0.05; **p < 0.01, ***p < 0.001 and ****p < 0.0001 indicates significant difference from OVA.

The effects of LRE on leukocyte infiltration in the peribronchiolar and perivascular region was also investigated. Prolonged OVA challenge caused a significant increase in the leukocyte infiltration throughout the study duration (Fig. 3). Following treatments, LRE125 significantly reduced leukocyte infiltration in the lung tissues after 2 and 10 weeks of the challenge when compared to OVA group. Besides, this study showed that after allergen cessation (week 12), treatment with LRE125 significantly reduced leukocyte infiltration in comparison to OVA group. No significant reduction to leukocyte infiltration following LRE treatments at 6 weeks of the challenge. Dexamethasone significantly attenuated leukocyte infiltration in Weeks 6, 10 and 12.

### The effect of LRE on goblet cell hyperplasia in the prolonged OVA-challenged allergic asthma model

**Figure 4:**
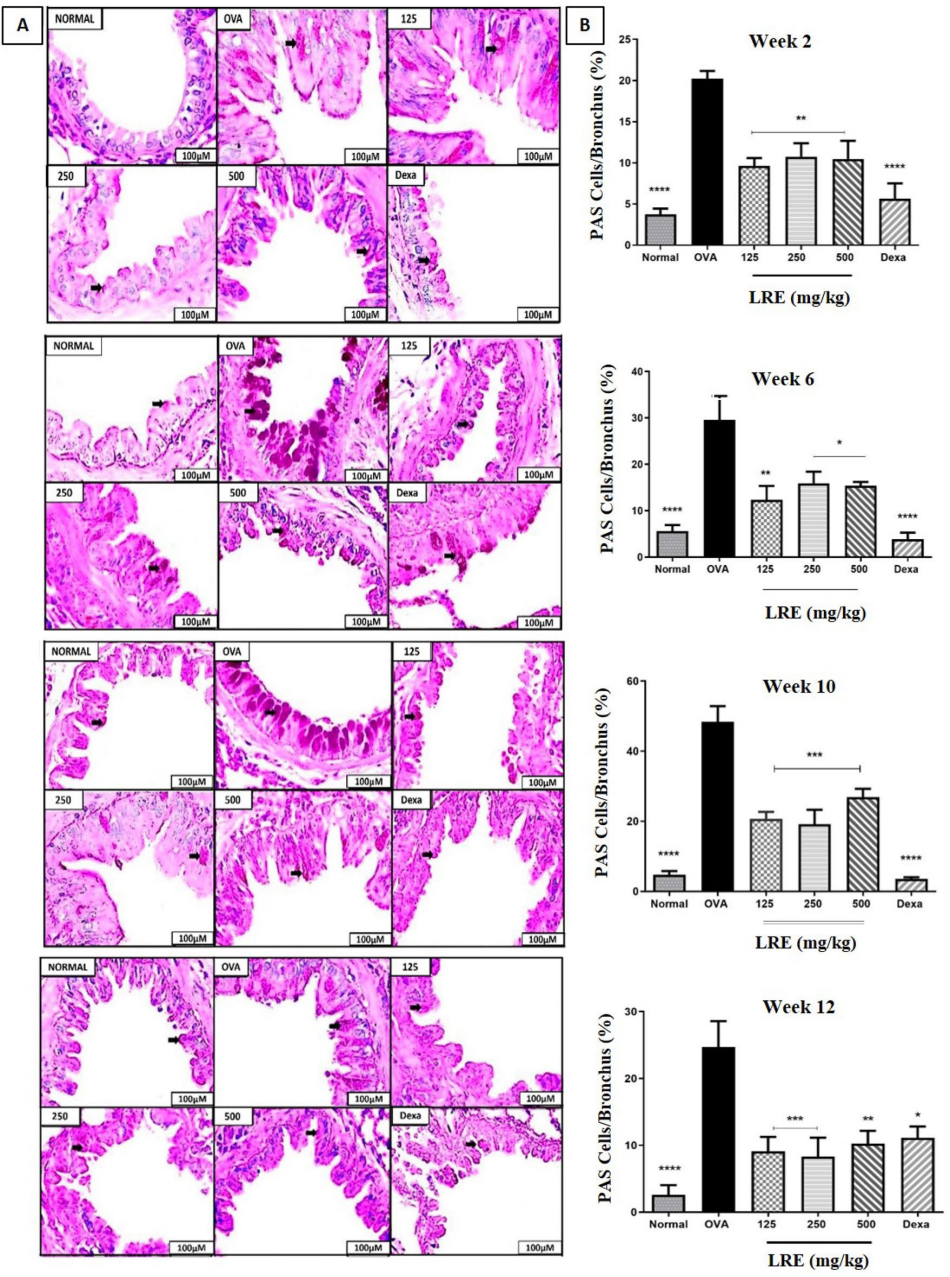
The effects of LRE on mucus production in prolonged OVA-challenged mice. (**A**) Representative photomicrographs OVA-sensitised/challenged mice; Normal; LRE125 mg/kg + OVA sensitised/ challenged mice; LRE250 mg/kg + OVA-sensitised/ challenged mice; LRE500 mg/kg + OVA-sensitised/ challenged mice; dexamethasone + OVA-sensitised/ challenged mice. Black arrows indicate the presence of stained goblet cells. All figures are in 20 × magnification. (**B**) Semi-quantitative analysis on the percentage of PAS-positive goblet cells using a numerical scoring system. Values are expressed as means ± SEM (n = 7 per group). OVA-group is significantly different from the normal group. *p < 0.05; **p < 0.01, ***p < 0.001 and ****p < 0.0001 indicates significant difference from OVA.

The effect of LRE on goblet cell hyperplasia in the prolonged OVA-challenged mice significantly developed goblet cell hyperplasia in the bronchi when compared to the normal group (Fig. 4). In this study, LRE-treated groups significantly attenuated the level of goblet cell hyperplasia when compared to OVA-group in Week 2, 6, 10 and 12. Similar observations were recorded in the lung tissues of Dexa group.

### The effects of LRE on airway smooth muscle deposition in the prolonged OVA-challenged allergic asthma model

**Figure 5:**
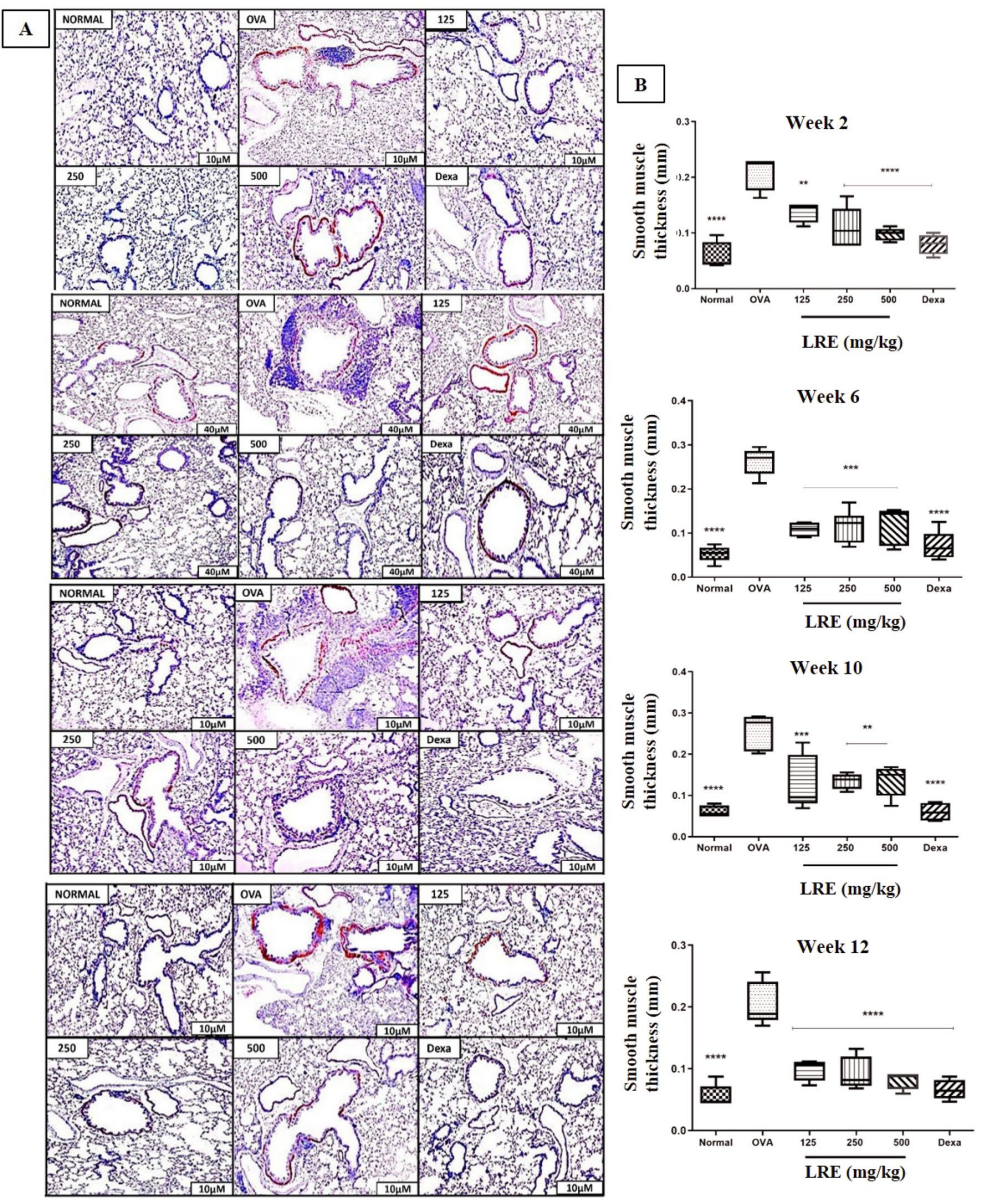
The effects of LRE on bronchial smooth muscle deposition in prolonged OVA-challenged mice. (**A**) Representative photomicrographs of α-smooth muscle actin (α-SMA)-stained. All figures are in 10× magnification. (**B**) Peribronchial smooth muscle deposition was quantified as the thickness of peribronchial α-SMA-stained layer. Values are expressed as means ± SEM (n = 7 per group). OVA-group is significantly different from the normal group. *p < 0.05; **p < 0.01, ***p < 0.001 and ****p < 0.0001 indicates significant difference from OVA.

Morphometric examination of airway section revealed that OVA-group significantly increased the ASM deposition throughout the study (Fig. 5). Treatment with dexamethasone significantly reduced the peribronchial smooth muscle deposition throughout the study. Similar observations were recorded by the LRE-treated groups, which demonstrated significant reduction of the thickness of peribronchial α-SMA-stained layer in Weeks 2, 6, 10 and 12 when compared to the OVA group.

### The effects of LRE on TGF-β1 and Activin A expressions in the lung tissues of prolonged OVA-challenged allergic asthma model

**Figure 6:**
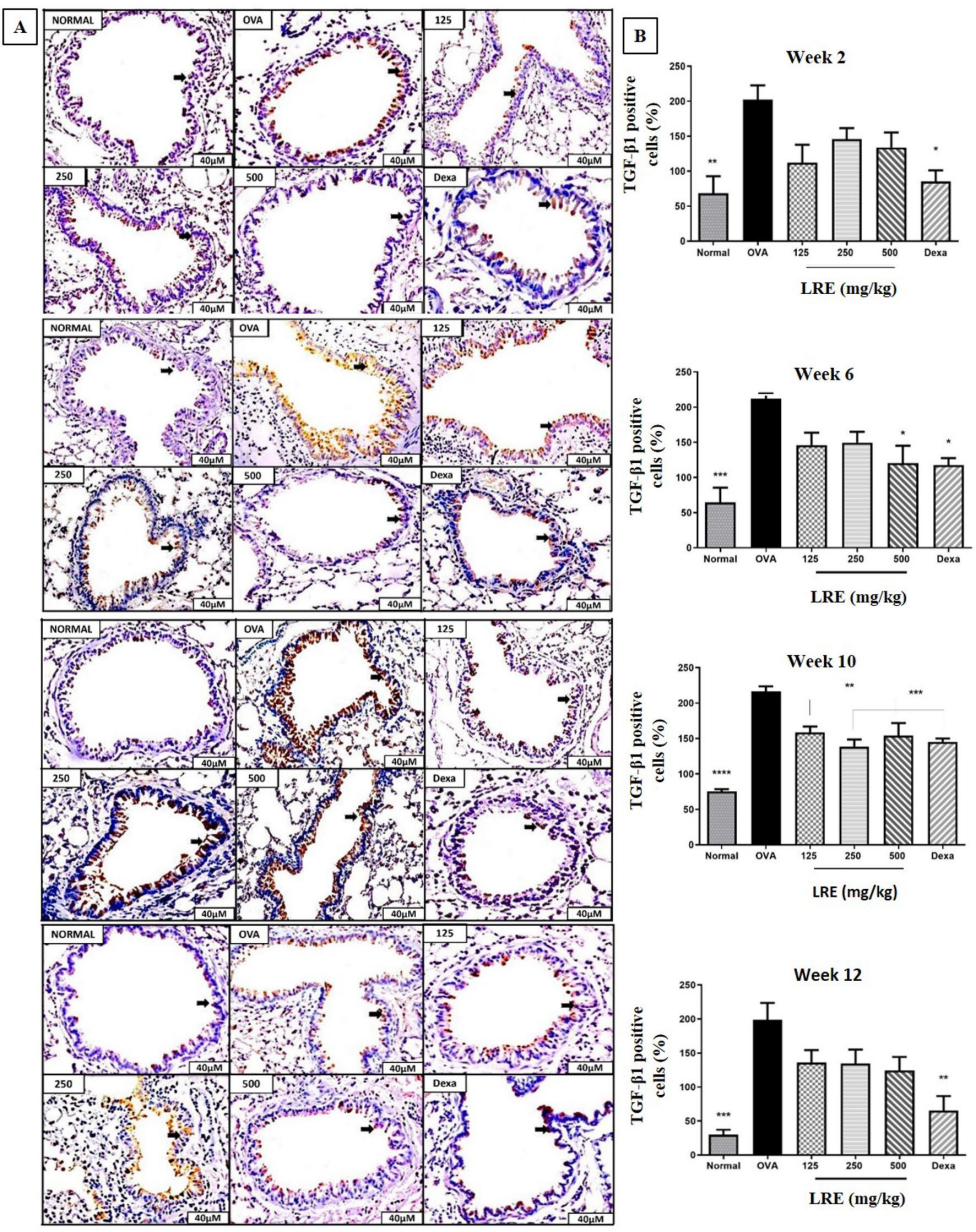
The effects of LRE on the expression of TGF-β1 in prolonged OVA-challenged mice. (**A**) Representative photomicrographs show OVA-sensitised/challenged mice; Normal; LRE125 mg/kg + OVA-sensitised/challenged mice; LRE250 mg/kg + OVA-sensitised/challenge mice; LRE500 mg/kg + OVA-sensitised/challenged mice; dexamethasone + OVA-sensitised/challenged mice. Brown color indicates the expression of TGF- β1 positive cells. All figures are in 40 × magnification. (**B**) Quantitative analysis of expression of TGF- β1 positive cells. Values are expressed as means ± SEM (n = 7 per group). OVA-group is significantly different from the normal group. *p < 0.05, **p < 0.01, ***p < 0.001 ****p < 0.0001and indicates significant difference from OVA.

Repetitive OVA-challenged induced a significant increase in the number of peribronchial transforming growth factor (TGF)-β1 positive cells in Week 2, Week 6, Week 10 and Week 12 compared to normal group (Fig. 6). Significant reduction of TGF-β1 expression was demonstrated by LRE500 group in Week 6; meanwhile, all LRE-treated groups significantly reduced TGF-β1 expression in Week 10. No significant reduction by any of LRE-treated groups in Weeks 2 and 12. Dexamethasone significantly reduced TGF-β1 expression throughout the study.

**Figure 7:**
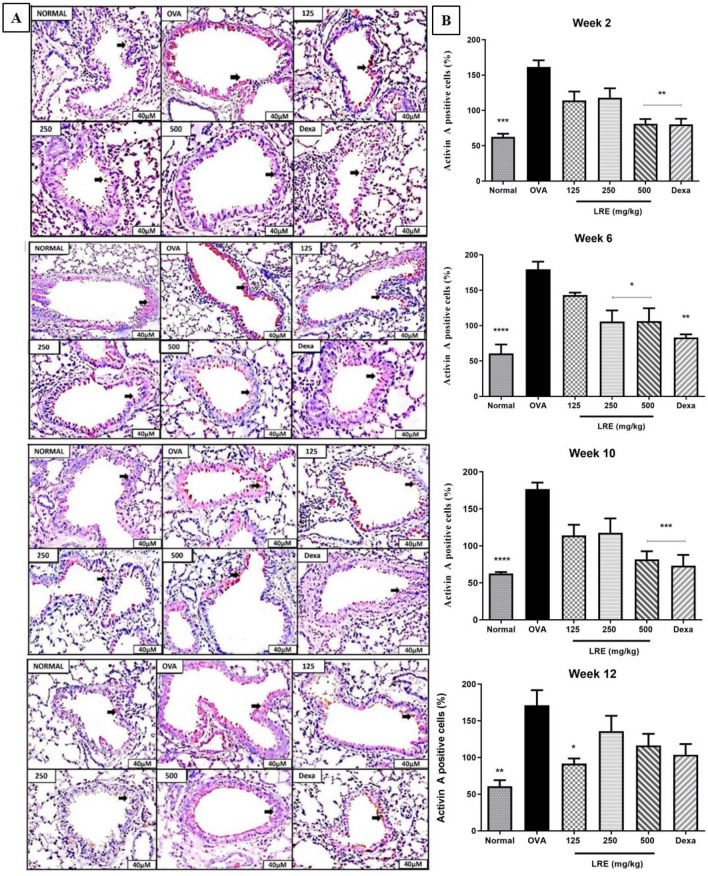
The effects of LRE on Activin A positive cell expression in prolonged OVA-challenged mice. (**A**) Representative photomicrographs show OVA-sensitised/challenged mice; Normal; LRE125 mg/kg + OVA-sensitised/challenged mice; LRE250 mg/kg + OVA-sensitised/challenged mice; LRE500 mg/kg + OVA sensitised/challenged mice; dexamethasone + OVA-sensitised/ challenged mice. Brown colour indicates the expression of Activin A positive cell. All figures are in 40× magnification. (**B**) Quantitative analysis of Activin A positive cell. Values are expressed as means ± SEM (n = 7 per group). OVA-group is significantly different from the normal group. *p < 0.05, **p < 0.01, ***p < 0.001 and ****p < 0.0001 indicates significantly difference from OVA.

Whereas, prolonged OVA challenge significantly increased the expression of Activin A positive cells in the OVA group throughout the study (Fig. 7). Following prolonged OVA challenge, LRE500 significantly attenuated the expression of Activin A positive cells in Weeks 2, 6 and 10. The LRE250 group also demonstrated significant attenuation of TGF-β1 expression in Week 6 and LRE125 in Week 12. Dexa group significantly reduced the expression of Activin A positive cell throughout the study.

## Discussion

Animal models can give a better understanding of the immune, respiratory system and their interaction in the lung^20^. However, it is vital to know that most animals used for asthmatic study do not spontaneously develop the disease, thus they must be sensitised and challenged with allergens in order to develop asthmatic-like immune responses^20^. Based on Mullane and Williams^21^, the protein ovalbumin (OVA) that derived from the egg white still become the first allergen of choice for asthmatic model. Repetitive OVA exposures via the airways elicit a Th-2 adaptive immune response leading to eosinophilia, goblet cell hyperplasia, airway hyperresposiveness^22, 23^ and pro-T2 adjuvant aluminum hydroxide (alum), helps in boosting the adaptive immune system via the inflammasome^24^. In the acute asthmatic model, the response could resolve spontaneously over a few days and these concerns have given impetus to the development of more chronic models. However, a limitation of animal models is that they cannot mimic all features and various phenotypes of the disease but can represent many inflammatory, structural and physiological features^25^.

LRE is traditionally used as a health tonic and for the treatment of chronic respiratory diseases such as asthma^26^. In the previous studies, LRE demonstrated anti-asthmatic effects by ameliorating some asthmatic parameters such as suppressing the level of Th2 cytokines in BALF, IgE in serum, leukocytes infiltration and mucus production in the lungs tissues^18, 19^ as well as AHR. In this study, the effect of LRE on prolonged OVA-challenged which mimicked the chronic asthma condition was evaluated. We demonstrated prolonged OVA induction significantly induced airway remodeling which were characterized by airway structural changes including goblet cell hyperplasia, increased smooth muscle mass; as well as increased the expression of α-SMA, activin A and TGF-β1. In the present study, LRE treatment inhibited airway inflammation and remodeling by significantly attenuating the parameters. This study indicated that the mechanism of airway remodeling suppression by LRE might be associated with the attenuation of α-SMA, activin A and TGF-β1 expressions.

Airway inflammation is the main feature of asthma; and the mechanism is mainly associated with various immune cells and inflammatory mediators. Among the immune cells, eosinophils is the major regulator of inflammation and remodeling in the asthmatic airway mucosa^27^. It has been reported that the degree of eosinophils infiltration was related to the disease severity, exacerbation frequency, deposition of collagen and proliferation of airway smooth muscle in chronic asthma^28^. In addition, alveolar macrophages play an important role in the late-phase inflammatory response of atopic asthmatic patients by enhancing the production of IL-5 by CD4 + T-cells, thereby increasing eosinophilic inflammation in the airways of asthmatic patients^29^. Our findings demonstrated that LRE significantly ameliorated airway inflammation in the prolonged OVA-challenged mice. These results suggested that amelioration of airway inflammation through the inhibition of inflammatory cells in BALF and eosinophil infiltration in the lung tissues might be associated with the anti-inflammatory mechanism of LRE. Treatments aimed at reducing eosinophilia in the airways have been successful in managing asthma symptoms^30, 31^.

In asthma, abnormal humoral and cellular immunological responses are the key elements involved in the pathophysiology of the disease^5, 32^. Aberrant expansion of Th2 cells and secretion of Th2 related cytokines play important roles in the manifestation of asthma^4^. In this study, a significant increase of the Th2 cytokines (IL-4, IL-5 and IL-13) levels were observed following prolonged OVA induction. Reducing the level of Th2 cytokines is crucial during allergic asthma responses which have been the target of many studies and are reported to be effective as anti-asthmatic therapy^33, 34^. IL-4 plays important roles in asthma, it induces Ig isotype switching and the production of IgE through the activation of B cells and promotes the production of eosinophil and neutrophil via mast cell stimulation^1^. Meanwhile, IL-5 promotes eosinophil survival, differentiation, and migration^1^. Whereas IL-13 stimulates IgE and eosinophil synthesis in a manner similar to IL-4; in addition, IL-13 also induces mucus metaplasia and AHR^35^. Thus, attenuation of Th2 cytokines is one of the effective strategies in asthma therapy. In the present study, LRE suppressed Th2 cytokines in the BALF and the IgE level in the serum, which indicated the effectiveness of LRE in ameliorating the airway inflammation in the prolonged OVA-challenged mice. Mucus hypersecretion is induced by airway inflammation when the number of goblet cells and the amount of mucin produced are significantly increased and results in airway obstruction in asthma^36^. Mucus hypersecretion related to an increased number of goblet cells which tends to be associated with severity of asthma^37^. The present study demonstrated LRE, and dexamethasone consistently inhibited goblet cell hyperplasia in the prolonged OVA-challenged mice. Similar effect was shown by Kamaruzaman et al.^38^ which demonstrated the effectiveness of aerosolised honey to reduce goblet cell hyperplasia and mucus hypersecretion in the asthma model. DEX is an oral corticosteroid that can be used in the treatment of patients affected by severe uncontrolled asthma when biological therapies are not indicated. Studies have shown that moderate DEX treatment reduces allergen-induced asthma models, reduces the expression of inflammatory factors in BALF and reduces infiltration of peribronchial inflammatory cells^39^.

Pathological changes in the airway wall and airway smooth muscle (ASM) layer thickening are important pathological features in asthmatic airway remodelling^40^. The increased ASM mass and increased airway wall thickness reduce airway lumen area, resulting in increased dynamic and fixed resistance^41–43^. A study by Halwani et al.^44^ reported that eosinophils promote airway remodelling during asthma by triggering ASM cell proliferation and thus increasing ASM mass. In addition, the Th2 cytokines, IL-4 and IL-13, are implicated in smooth muscle hyperplasia^45^. In this study, treatment with LRE (125, 250 and 500 mg/kg) significantly reduced the thickness of ASM in comparison to OVA-challenged group throughout the weeks. This is in comparison with the IL-4 and IL-13 level where the reduction of these cytokines is significant in lowest LRE treatment of 125 mg/kg. These results demonstrated that the administration of LRE could reduce smooth muscle thickness which is important in improving airway remodelling. It was in agreement with the study by Al-Muhsen et al. and Prakash which stated that reduction of ASM mediated by chrysin (a natural flavonoid) might be partly due to its impact on cytokines and inflammatory cells as well as its direct action of ASM cells itself^41, 46^. Moreover, the present finding was in agreement with Zeng et al.^47^, which stated that the administration of curcumin reduces the thickening of the airway wall and bronchial smooth muscle layer. This result further indicates that LRE demonstrates a therapeutic effect in altering airway remodelling.

TGF-β1 is a profibrotic cytokine implicated in the pathogenesis of asthma. TGF-β1 in asthma causes epithelial transformation, subepithelial fibrosis, remodelling of ASM, microvascular changes, and development of mucus in the lung tissues^48, 49^. Previous studies have shown the presence of TGF-ß1-positive cells in the bronchial biopsies of severe asthma^50–52^. Meanwhile, activin A is a pleiotropic cytokine and a member of the TGF-β superfamily, with a central role in inflammation and tissue remodelling^53, 54^. The increased expression of activin A in clinical and experimental studies on asthma suggests that this cytokine is involved in the pathogenesis of asthma^55^. A previous study by Gregory et al. (2010) demonstrated overexpression of the TGF-β/activin signalling intermediate Smad2 in airway epithelium induced activin A secretion and airway remodelling^56^. In this study, TGF-β1 and activin A were expressed in the lung tissues after 2 weeks of OVA-challenged and remained highly expressed during prolonged OVA-challenged at Week 6 and 10 but slightly decreased once OVA-challenged ceased. These changes were in line with the previous study by Hardy et al.^57^, which suggested that decreasing levels of TGF-β1 and Activin A following allergen cessation were related with the resolution of airway remodelling. Here, there were significant reductions of TGF-β1 and Activin A positive cells in mice of the prolonged OVA-challenged by the selected LRE treatments. This study revealed that LRE and dexamethasone treatments could reverse the expression of TGF-β1, and this alleviation might be responsible for the reversal of airway remodelling, including the attenuation of mucus secretion and collagen production. Our result corresponds well with^58^, which suggested that treatment with Astragalus extract reduced the expression of TGF-β1, TGF-β1 mRNA, and modulated active TGF-β1 signalling in the airways. This study has concluded that decrease of TGF-β1 levels and modulation of the TGF-β1 signalling pathway is a possible mechanism by which the Astragalus extract inhibited airway remodelling in asthma. 

Interestingly, the inhibitory effect of LRE was comparable to the standard drug, dexamethasone which significantly reduced the recruitment of inflammatory cells over the prolonged allergen challenged. It showed dexamethasone inhibited hallmark features of eosinophilic asthma, including Th2 production, IgE synthesis and airway remodelling. Thus, administration of LRE or dexamethasone were effective in significantly inhibiting the recruitment of leukocyte infiltration in the airways and inhibited peribronchiolar inflammation in prolonged allergen exposure. The present study shows that LRE possesses a therapeutic anti-asthmatic potential by ameliorating the inflammation and remodelling of the airway. To date, this is the first study that reported the effect of LRE on airway remodelling in allergic asthma by targeting TGF-β1 and activin A. Thus, further study on active constituents in the LRE is needed prior to formulation of the extract for the allergic asthma use in the future study.

## Methods

### *L. rhinocerotis* extraction

Cultivated *L. rhinocerotis* sclerotia powder, TM02 cultivar, was obtained from Ligno Biotech Sdn. Bhd. Formal identification of the mushroom was performed by Dr. Tan Chon Seng from the Institute of Agricultural Research and Development Malaysia. According to Tan et al.^59^, the mushroom was authenticated by the internal transcribed spacer (ITS) regions of their ribosomal RNA. The mushroom specimen voucher was deposited at the Royal Botanic Garden Kew, with the accession no. K(M) 177,812. The study was conducted in accordance with the Guideline for Herbal Medicine Research from the National Committee for Research and Development of Herbal Medicine (NRDHM), Ministry of Health Malaysia. In this study, the mushroom was subjected to hot water extraction using a soxhlet extractor^60^. 50 g of *L. rhinocerotis* powder was inserted into an extraction thimble (22 mm internal diameter and 90 mm external length) and 600 ml of purified distilled water (dH2O) was used as a solvent. The extraction was performed for 24 h, followed by extract filtration using filter paper before being concentrated with a rotary evaporator (Ilshin BioBase, Gyeonggi-do, Korea). The extract was freeze-dried into lyophilised powder form for 36 h. Approximately 5 g of *L. rhinocerotis* extract could be obtained from 50 g of sclerotial powder (10% yield).

### LRE and drug preparation for animal procedure

LRE was dissolved in phosphate buffer saline (PBS) and the required dose of LRE (125, 250 and 500 mg/kg) were calculated based on the mice body weight as follow:

$${\text{Calculated}}\;{\text{injected}}\;{\text{volume}}\left( {{\text{ml}}} \right) = \frac{{{\text{Animal}}\;{\text{weight}}\left( {{\text{kg}}} \right) \times {\text{Dose}}\left( {{\text{mg}}/{\text{kg}}} \right)}}{{{\text{Concentration}}\;\left( {{\text{mg}}/{\text{ml}}} \right)}}$$

Similar preparation was carried out for dexamethasone (3 mg/kg) which was used as a positive control. Both LRE and dexamethasone were given orally by gavage [depositing LRE into the esophagus using a syringe with gavage needle (blunt-ended needle cannula)] and by i.p injection respectively.

### Experimental protocols

**Figure 8:**
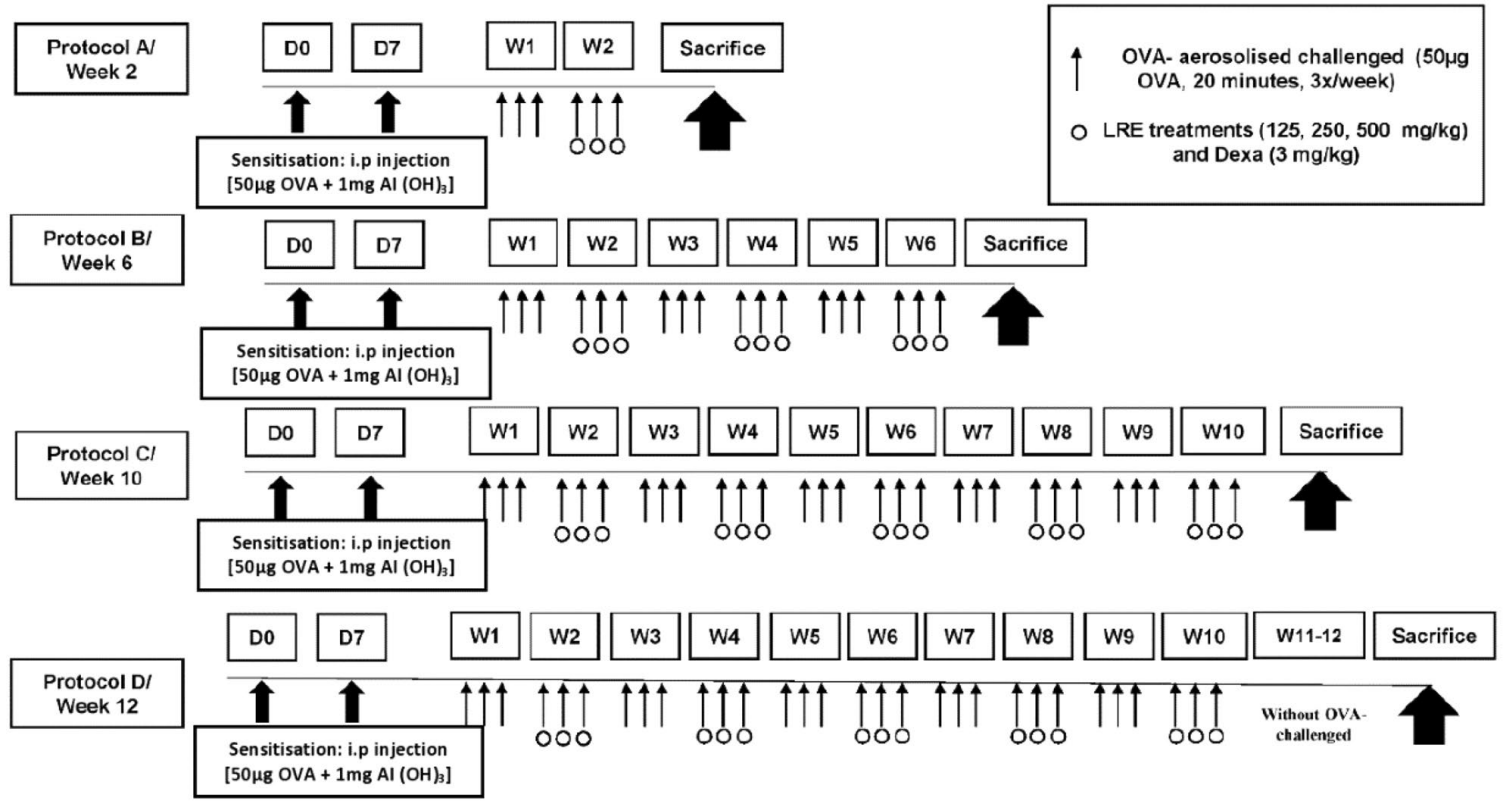
Sensitisation, challenge and treatment protocols for prolonged OVA-challenged mouse model of asthma. Protocols (**A**) 2 weeks; (**B**) 6 weeks; (**C**) 10 weeks; (**D**) 12 weeks models of OVA-challenged. Mice were sensitised on day 0 and 7 by intraperitoneal (i.p) injection of a mixture containing 50 µg of ovalbumin and 1 mg of aluminium hydroxide [Al(OH)3]. The mice were challenged with 50 µg of OVA for 3 times per week followed by treatments (LRE and dexamethasone).

Healthy female Balb/c mice, age 6–8 weeks were obtained from Universiti Putra Malaysia (UPM) Serdang, Malaysia and housed in the Animal Research and Service Centre (ARASC), Universiti Sains Malaysia (USM). The animal care and experimental protocols were reviewed and approved by the Universiti Sains Malaysia Institutional Animal Care and Use Committee (USM/Animal Ethics Approval/2015/(97)(687)). The experimental procedures conformed to the Animal Research: Reporting In Vivo Experiments (ARRIVE) guidelines for animal experiments. The animals were housed in specialized polypropylene cages with free excess to normal standard food pellet supplied by ARASC USM and water ad libitum under standard laboratory conditions, which included a 12-h light/dark schedule, a temperature of 23 ± 2 °C and a humidity within 50–60%. The mice were acclimatised for seven days to the experimental environment before the commencement of the study. The mice were randomly divided into six groups, comprising of seven mice per group (n = 7): (1) normal group (2) OVA, sensitised and challenged with 1% ovalbumin (OVA) (3) LRE125, sensitised and challenged with OVA; treated with oral LRE (125 mg/kg) (4) LRE250, sensitised and challenged with OVA; treated with oral LRE (250 mg/kg) (5) LRE500, sensitised and challenged with OVA; treated with oral LRE (500 mg/kg) (4) Dex, sensitised and challenged with OVA; treated with dexamethasone (3 mg/kg). The mice were sensitised on day 0 and 7 mice with a mixture of 50 µg of ovalbumin (OVA; Grade V, Sigma, USA) with 1 mg of aluminium hydroxide [Al(OH)3] (Nacalai Tesque, Japan) in 1 × phosphate buffer solution (PBS) (a total volume of 0.2 ml/mouse) by intraperitoneal (i.p) injection as described by Stumm et al., (2014). Then, on day 14, the mice were challenged by aerosolised-OVA (50 µg) three times per week and the duration was 20 min/session. The aerosol exposure was conducted within a chamber that was coupled to an ultrasonic nebulizer (Mabist mist; Mabist DMI Healthcare, Illinois, CA, USA), wherein the mice were positioned within the chamber, and subsequently, the nebuliser was activated to aerosolise the OVA solution from the nebuliser cup. Then, 100 µl LRE treatments (125 mg/kg, 250 mg/kg and 500 mg/kg) were administered into the mice respectively via oral gavage one hour after the OVA challenge, on an alternating weekly basis. A normal control group was administered PBS instead of OVA. The mice were euthanised after 2, 6 and 10 weeks of OVA challenges, as well as two weeks after the final challenge (12 weeks), using 200 mg/kg pentobarbitone administered peritoneally (see Fig. 8).

### Collection of bronchoalveolar lavage fluid (BALF) and leukocyte counts

Twenty-four hours after the final challenge, BALF was obtained from the mice trachea using an endotracheal tube by instillation and aspiration using 0.4 ml of 1% fetal calf serum (FCS) in PBS for three times (total up to 1.2 ml). The BALF was centrifuged at 350 × g for 5 min at 4 °C, and the supernatant was collected and stored at -80 °C for cytokine analysis, while BALF pellet was used for total cell count. The cell pellets were re-suspended in 100 µl of sterile PBS, and cyto-centrifugation was then carried out using cytospin centrifuge (Thermo Shando, Pittsburgh, USA). The cells were air-dried and stained with methanol for 1–2 min, followed by Giemsa staining (Merck, Germany) for 8 min, and the dried slides were mounted. A minimum of 200 inflammatory cells were identified by standard morphology criteria using a light microscope (Olympus, USA) at 100 × magnification. The relative count number of eosinophil, macrophage, lymphocyte and neutrophil were calculated.

### Measurement of T helper 2 (Th2) cytokines in BALF

Levels of IL-4, IL-5 and IL-13 in BALF were measured using specific mouse IL-4, IL-5 (Biolegend, USA) and IL-13 (Peprotech, USA) ELISA kits. 100 µl diluted capture antibody solutions were added into 96-well plate, sealed, and incubated overnight (16–18 h) at 4 °C. On the next day, the wells were washed and blocked with (200 µl of 1 × assay diluent for IL-4 and IL-5; 300 µl blocking buffer for IL-13) for one hour at RT. After blocking, 100 µl of the diluted standards and samples (BALF supernatant) were loaded, incubated for 2 h, washed, and incubated with 100 µl of diluted detection antibody solution, followed by diluted Avidin-HRP for 30 min. After final wash, the wells were soaked in wash buffer for 30 s to one minute for each wash to help in minimizing the background, followed by addition of 100 µl of freshly mixed 3,3',5,5' Tetramethylbenzidine (TMB) substrate solution before adding 100 µl of stop solution to each well. The optical density (OD) of solution was read at 450 nm with correction at 570 nm. Cytokine concentrations were calculated from standard curves that were generated using respective standards.

### Measurement of total IgE in serum

Following sacrifice, 0.5–0.7 ml of blood was collected by cardiac puncture. Blood was then allowed to clot for at least three hours at the RT before centrifuged at 350 × g for 10 min at 4 °C. The serum was extracted carefully and stored at -80 °C for subsequent experiments. The level of total IgE in serum was measured with a specific mouse ELISA kit (BD Biosciences, San Diego, USA) according to the manufacturer’s instructions. 96 well-ELISA plate was coated with 100 μl diluted capture antibody, incubated overnight, washed, and blocked with 200 μl of assay diluents for 1 h at RT. Then 100 μl of diluted standards and serum samples (1:100) were loaded, and incubated for 2 h at RT. Following washing, 100 μl detection antibody was added and incubated with substrate solution in dark condition for 15–30 min. Finally, to stop the reaction, 50 μl of stop solution was added and the OD readings was read at 450 nm, with correction at 570 nm. Results were analysed by SkanIt Software 2.4.3 RE and a linear standard curve was generated between 0 and 10 ng/ml.

### Lung Histopathology

After sacrifice, lung tissues were fixed in 10% formalin overnight, dehydrated using ethanol and embedded in paraffin. The tissue Sections (3.5 μm) were deparaffinised in xylene, rehydrated in a graded series of ethanol solutions, and stained with hematoxylin and eosin (H&E) (Sigma Aldrich, USA) and Periodic acid-Schiff (PAS) (Sigma Aldrich, USA). For H&E staining, the infiltration intensity at the peribronchial and perivascular inflammatory spaces was scored as the number of cell layers around the bronchioles, following the criteria adapted from Myou et al.^61^. The severity of inflammation was graded semi-quantitatively using the numerical scoring system according to these features; 0: no inflammatory cells, 1: a few inflammatory cells, 2: a ring of inflammatory cells (1 cell layer deep), 3: a ring of inflammatory cells (2–4 cells layer deep) and 4: a ring of inflammatory cells (> 4 cells layer deep).

For PAS staining, the presence of PAS-positive goblet cells in the airway epithelium was graded semi-quantitatively using the numerical scoring system^61^ following these features: 0 (no goblet cells); 1 (< 25% of epithelium); 2 (25–50% of epithelium); 3 (50–75% of epithelium);4 (> 75% of epithelium). Both H&E and PAS staining were scored by double-blind scoring method with 10% of the slides randomly picked by a pathologist to confirm the observation. Images were captured using an inverted microscope (Olympus, USA) and semi-quantitative analysis was performed.

### Lung immunohistochemistry

For both α-smooth muscle actin (α-SMA) and transforming growth factor-β1 (TGF-β1), mice lung tissue sections were fixed, deparaffinized, dehydrated through an ascending ethanol series (80%, 95% (2x) and 100% (3x), heated in pressure cooker (121 °C) containing 1L of pH citrate buffer for 10 min and allowed to cool at room temperature for 5 min before blocking by endogenous peroxidase for 5 min and washed. The specimens were incubated overnight at 4 °C with a biotinylated antibody containing α-SMA monoclonal antibody (Novusbio, USA), biotinylation reagent, blocking reagent and diluent (Envision system; DAKO, Germany) at a dilution of 1:300 and TGF-β1 monoclonal antibody (Novusbio, USA) at a dilution of 1:50. Substitution of the primary antibody with tris buffer saline in 0.1% tween 20 (TBS-T) was used separately as a negative control.

For Activin A, tissue slides were blocked by peroxidase blocking reagent (3% of hydrogen peroxidase) for 5 min, washed and overnight incubated with serum blocking reagent G (for mouse) (R&D system, USA) at 4 °C before incubated with Avidin blocking reagent and biotin blocking reagent for 15 min respectively. After washing, the slides were incubated with mouse Activin A βA subunit antibody (R&D system) at a dilution of 1:40 for an hour, while negative control slides were incubated with PBS. After a series of washing, the slides were incubated with biotinylated secondary antibody (anti-mouse secondary antibody in 0.01 M PBS containing 0.1% NaN3) and HSS-HRP (R&D system, USA) for 30 min at RT. After that, the slides were washed and stained with DAB chromogen (Envision system; DAKO, Germany) for 5 min, counterstained with haematoxylin for 10 s and dehydrated with series of alcohol and subsequently mounted with DPX (Merck, Germany).

According to Cho et al.^62^, the ASM deposition was quantified as the thickness of peribronchial α-SMA-stained layer using Image J software. Each bronchiole was measured at four different places and the average score was used for statistical analysis.

Meanwhile, expression of TGF-β1 and Activin-A were performed by using the H-score method with blinded scoring (Mazières et al., 2013). Scores from 0, 1, 2 and 3 represented negative, weak, moderate, and strong expressions of the stained cells respectively. Each score was obtained by using formula; (0 × negatively stained %) + (1 × weakly stained %) + (2 × moderately stained %) + (3 × strongly stained %), ranging total score from 0 to 300. Increasing total range number indicates increasing expression of the stained around the whole bronchiole.

### Statistical analysis

Statistical analysis of the data set obtained in this study was analysed using Graph Pad Prism Version 6.0. Data were analysed for normality, and all data were expressed as the mean ± standard error of the mean (SEM). Comparisons between untreated (OVA) and treatment groups were conducted using one-way analysis of variance (ANOVA) followed by Bonferroni’s test. The critical level for statistical significance was set at *p* < 0.05.

### Ethics approval

 The animal care and experimental protocols in the study were reviewed and approved by the Universiti Sains Malaysia Institutional Animal Care and Use Committee (USM/Animal Ethics Approval/2015/(97)(687)). The experimental procedures conformed to the Animal Research: Reporting In Vivo Experiments (ARRIVE) guidelines for animal experiments.

## Data Availability

The research study includes the original contributions, and any additional inquiries can be directed to the corresponding author.

## References

[1] Lambrecht BN, Hammad H, Fahy JV (2019). The cytokines of asthma. Immunity.

[2] Agarwal R, Gupta D (2011). Severe asthma and fungi: Current evidence. Med. Mycol..

[3] Network, G. A. Global Asthma Report 2014. **769**, 28–36 (2014).

[4] Fahy JV (2015). Type 2 inflammation in asthma—Present in most, absent in many. Nat. Rev. Immunol..

[5] Lambrecht BN, Hammad H (2015). The immunology of asthma. Nat. Immunol..

[6] Barnes PJ (2018). Targeting cytokines to treat asthma and chronic obstructive pulmonary disease. Nat. Rev. Immunol..

[7] Fehrenbach H, Wagner C, Wegmann M (2017). Airway remodeling in asthma: What really matters. Cell Tissue Res..

[8] Bai TR (2010). Evidence for airway remodeling in chronic asthma. Curr. Opin. Allergy Clin. Immunol..

[9] Hirota N, Martin JG (2013). Mechanisms of airway remodeling. Chest.

[10] Tai A (2014). The association between childhood asthma and adult chronic obstructive pulmonary disease. Thorax.

[11] Bergeron C, Tulic MK, Hamid Q (2010). Airway remodelling in asthma: From benchside to clinical practice. Can. Respir. J..

[12] Boskabady MH, Tabatabaee A, Byrami G (2012). The effect of the extract of *Crocus sativus* and its constituent safranal, on lung pathology and lung inflammation of ovalbumin sensitized guinea-pigs. Phytomedicine.

[13] Boskabady MH, Shahmohammadi Mehrjardi S, Rezaee A, Rafatpanah H, Jalali S (2013). The impact of *Zataria multiflora* Boiss extract on in vitro and in vivo Th1/Th2 cytokine (IFN-γ/IL4) balance. J. Ethnopharmacol..

[14] Kianmehr M (2017). The effect of *Zataria multiflora* on Th1/Th2 and Th17/T regulatory in a mouse model of allergic asthma. Front. Pharmacol..

[15] Wong K-H, Lai CKM, Cheung PCK (2011). Immunomodulatory activities of mushroom sclerotial polysaccharides. Food Hydrocoll..

[16] Yap YH (2013). Nutrient composition, antioxidant properties, and anti-proliferative activity of *Lignosus rhinocerus* Cooke sclerotium. J. Sci. Food Agric..

[17] Lee SS (2014). Anti-inflammatory effect of the sclerotium of *Lignosus rhinocerotis* (Cooke) Ryvarden, the Tiger Milk mushroom. BMC Complement. Altern. Med..

[18] Johnathan M, Gan SH, Ezumi MFW, Faezahtul AH, Nurul AA (2016). Phytochemical profiles and inhibitory effects of Tiger Milk mushroom (*Lignosus rhinocerus*) extract on ovalbumin-induced airway inflammation in a rodent model of asthma. BMC Complement. Altern. Med..

[19] Muhamad S (2019). Intranasal administration of *Lignosus rhinocerotis* (Cooke) Ryvarden (Tiger Milk mushroom) extract attenuates airway inflammation in murine model of allergic asthma. Exp. Ther. Med..

[20] Van der Velden J, Snibson KJ (2011). Airway disease: The use of large animal models for drug discovery. Pulm. Pharmacol. Ther..

[21] Mullane K, Williams M (2014). Animal models of asthma: Reprise or reboot?. Biochem. Pharmacol..

[22] Alessandrini F (2010). Effects of ultrafine particles-induced oxidative stress on Clara cells in allergic lung inflammation. Part. Fibre Toxicol..

[23] Debeuf N, Haspeslagh E, Helden M, Hammad H, Lambrecht BN (2016). Mouse models of asthma. Curr. Protoc. Mouse Biol..

[24] Kool M (2008). Cutting edge: Alum adjuvant stimulates inflammatory dendritic cells through activation of the NALP3 inflammasome. J. Immunol..

[25] Chapman DG, Tully JE, Nolin JD, Janssen-Heininger YM, Irvin CG (2014). Animal models of allergic airways disease: Where are we and where to next?. J. Cell. Biochem..

[26] Lau BF, Abdullah N, Aminudin N, Lee HB, Tan PJ (2015). Ethnomedicinal uses, pharmacological activities, and cultivation of Lignosus spp. (tiger׳s milk mushrooms) in Malaysia—A review. J. Ethnopharmacol..

[27] Murdoch JR, Lloyd CM (2010). Chronic inflammation and asthma. Mutat. Res./Fundam. Mol. Mech. Mutagen..

[28] Price DB (2015). Blood eosinophil count and prospective annual asthma disease burden: a UK cohort study. Lancet Respir. Med..

[29] Fricker M, Gibson PG (2017). Macrophage dysfunction in the pathogenesis and treatment of asthma. Eur. Respir. J..

[30] Steinke JW, Lawrence MG (2014). T-cell biology in immunotherapy. Ann. Allergy Asthma Immunol..

[31] Fulkerson PC, Rothenberg ME (2013). Targeting eosinophils in allergy, inflammation and beyond. Nat. Rev. Drug Discov..

[32] Deckers J, Branco-Madeira F, Hammad H (2013). Innate immune cells in asthma. Trends Immunol..

[33] Lee J-H (2010). Type I IL-1 receptor (IL-1RI) as potential new therapeutic target for bronchial asthma. Mediat. Inflamm..

[34] Mahajan SG, Mehta AA (2011). Suppression of ovalbumin-induced Th2-driven airway inflammation by β-sitosterol in a guinea pig model of asthma. Eur. J. Pharmacol..

[35] Larché M, Robinson DS, Kay AB (2003). The role of T lymphocytes in the pathogenesis of asthma. J. Allergy Clin. Immunol..

[36] Schuijs MJ, Willart MA, Hammad H, Lambrecht BN (2013). Cytokine targets in airway inflammation. Curr. Opin. Pharmacol..

[37] Shen Y (2018). Management of airway mucus hypersecretion in chronic airway inflammatory disease: Chinese expert consensus (English edition). Int. J. Chronic Obstr. Pulm. Dis..

[38] Kamaruzaman NA, Sulaiman SA, Kaur G, Yahaya B (2014). Inhalation of honey reduces airway inflammation and histopathological changes in a rabbit model of ovalbumin-induced chronic asthma. BMC Complement. Altern. Med..

[39] Guan M (2020). Dexamethasone alleviate allergic airway inflammation in mice by inhibiting the activation of NLRP3 inflammasome. Int. Immunopharmacol..

[40] Rhee CK (2011). Effect of imatinib on airway smooth muscle thickening in a murine model of chronic asthma. Int. Arch. Allergy Immunol..

[41] Prakash YS (2013). Airway smooth muscle in airway reactivity and remodeling: What have we learned?. Am. J. Physiol. Lung Cell. Mol. Physiol..

[42] Nayak AP, Deshpande DA, Penn RB (2018). New targets for resolution of airway remodeling in obstructive lung diseases. F1000Research.

[43] Gosens R, Grainge C (2015). Bronchoconstriction and airway biology. Chest.

[44] Halwani R (2013). eosinophils induce airway smooth muscle cell proliferation. J. Clin. Immunol..

[45] Nath P (2007). Complete inhibition of allergic airway inflammation and remodelling in quadruple IL-4/5/9/13-/- mice. Clin. Exp. Allergy.

[46] Al-Muhsen S, Johnson JR, Hamid Q (2011). Remodeling in asthma. J. Allergy Clin. Immunol..

[47] Zeng X (2013). Curcumin inhibits the proliferation of airway smooth muscle cells in vitro and in vivo. Int. J. Mol. Med..

[48] Al-Alawi M, Hassan T, Chotirmall SH (2014). Transforming growth factor β and severe asthma: A perfect storm. Respir. Med..

[49] Halwani R, Al-Muhsen S, Al-Jahdali H, Hamid Q (2011). Role of transforming growth factor–β in airway remodeling in asthma. Am. J. Respir. Cell Mol. Biol..

[50] Vignola AM (1997). Transforming growth factor- β expression in mucosal biopsies in asthma and chronic bronchitis. Am. J. Respir. Crit. Care Med..

[51] Minshall EM (1997). Eosinophil-associated TGF- β 1 mRNA expression and airways fibrosis in bronchial asthma. Am. J. Respir. Cell Mol. Biol..

[52] Ohno I (1996). Transforming growth factor beta 1 (TGF beta 1) gene expression by eosinophils in asthmatic airway inflammation. Am. J. Respir. Cell Mol. Biol..

[53] Forrester HB (2013). Follistatin is induced by ionizing radiation and potentially predictive of radiosensitivity in radiation-induced fibrosis patient derived fibroblasts. PLoS One.

[54] de Kretser DM, O’Hehir RE, Hardy CL, Hedger MP (2012). The roles of activin A and its binding protein, follistatin, in inflammation and tissue repair. Mol. Cell. Endocrinol..

[55] Kariyawasam HH, Semitekolou M, Robinson DS, Xanthou G (2011). Activin-A: A novel critical regulator of allergic asthma. Clin. Exp. Allergy.

[56] Gregory LG (2010). Overexpression of Smad2 drives house dust mite–mediated airway remodeling and airway hyperresponsiveness via activin and IL-25. Am. J. Respir. Crit. Care Med..

[57] Hardy CL (2013). The activin A antagonist follistatin inhibits asthmatic airway remodelling. Thorax.

[58] Qu Z-H (2012). Inhibition airway remodeling and transforming growth factor-β1/Smad signaling pathway by astragalus extract in asthmatic mice. Int. J. Mol. Med..

[59] Tan CS, Ng ST, Vikineswary S, Lo FP, Tee CS (2010). Genetic markers for identification of a malaysian medicinal mushroom, *Lignosus rhinocerus* (Cendawan Susu Rimau). Acta Hortic..

[60] Gbolagade JS (2007). Antagonistic effect of extracts of some Nigerian higher fungi against selected pathogenic microorganisms. Am.-Eurasian J. Agric. Environ. Sci..

[61] Myou S (2003). Blockade of Inflammation and airway hyperresponsiveness in immune-sensitized mice by dominant-negative phosphoinositide 3-kinase–TAT. J. Exp. Med..

[62] Cho JY (2004). Inhibition of airway remodeling in IL-5–deficient mice. J. Clin. Investig..

